# The effect of growth rate on pyrazinamide activity in *Mycobacterium tuberculosis* - insights for early bactericidal activity?

**DOI:** 10.1186/s12879-016-1533-z

**Published:** 2016-05-17

**Authors:** Steven T. Pullan, Jon C. Allnutt, Rebecca Devine, Kim A. Hatch, Rose E. Jeeves, Charlotte L. Hendon-Dunn, Philip D. Marsh, Joanna Bacon

**Affiliations:** Public Health England, National Infection Service, Porton Down, Salisbury, Wiltshire SP4 0JG UK; School of Biological Sciences, University of East Anglia, Norwich Research Park, NR4 7TJ UK

**Keywords:** Pyrazinamide, Chemostats, Growth rate, Gene expression, *trans*-translation, *Mycobacterium tuberculosis*

## Abstract

**Background:**

Pyrazinamide (PZA) plays an essential part in the shortened six-month tuberculosis (TB) treatment course due to its activity against slow-growing and non-replicating organisms. We tested whether PZA preferentially targets slow growing cells of *Mycobacterium tuberculosis* that could be representative of bacteria that remain after the initial kill with isoniazid (INH), by observing the response of either slow growing or fast growing bacilli to differing concentrations of PZA.

**Methods:**

*M. tuberculosis* H37Rv was grown in continuous culture at either a constant fast growth rate (Mean Generation Time (MGT) of 23.1 h) or slow growth rate (69.3 h MGT) at a controlled dissolved oxygen tension of 10 % and a controlled acidity at pH 6.3 ± 0.1. Cultures were exposed to step-wise increases in the concentration of PZA (25 to 500 μgml^−1^) every two MGTs, and bacterial survival was measured. PZA-induced global gene expression was explored for each increase in PZA-concentration, using DNA microarray.

**Results:**

At a constant pH 6.3, actively dividing mycobacteria were susceptible to PZA, with similar responses to increasing concentrations of PZA at both growth rates. Three distinct phases of drug response could be distingished for both slow growing (69.3 h MGT) and fast growing (23.1 h MGT) bacilli. A bacteriostatic phase at a low concentration of PZA was followed by a recovery period in which the culture adapted to the presence of PZA and bacteria were actively dividing in steady-state. In contrast, there was a rapid loss of viability at bactericidal concentrations. There was a notable delay in the onset of the recovery period in quickly dividing cells compared with those dividing more slowly. Fast growers and slow growers adapted to PZA-exposure via very similar mechanisms; through reduced gene expression of tRNA, 50S, and 30S ribosomal proteins.

**Conclusions:**

PZA had an equivalent level of activity against fast growing and slow growing *M. tuberculosis.* At both growth rates drug-tolerance to sub-lethal concentrations may have been due to reduced expression of tRNA, 50S, and 30S ribosomal proteins. The findings from this study show that PZA has utility against more than one phenotypic sub-population of bacilli and could be re-assessed for its early bactericidal activity, in combination with other drugs, during TB treatment.

**Electronic supplementary material:**

The online version of this article (doi:10.1186/s12879-016-1533-z) contains supplementary material, which is available to authorized users.

## Background

An important aim for improving TB treatment is to shorten the period of antibiotic therapy without increasing relapse rates or encouraging the development of antibiotic-resistant strains. Pyrazinamide (PZA) is a key component of front-line chemotherapy against *Mycobacterium tuberculosis* (*M. tuberculosis*). It is thought to play an essential role in the shortened 6-month treatment course [1, 2] due to its ability to act upon the slow-growing drug-tolerant organisms or antibiotic resistant organisms that emerge following treatment with the other front-line drugs, isoniazid (INH) and rifampicin.

In vitro, PZA is still far less potent than in vivo [3] and its activity against actively growing bacteria in buffered growth media has been found to be extremely low, but increases against stationary phase bacteria either under acidic conditions or if the expression of PZA-activating enzyme, PncA, is artificially increased [4]. Previously, Zhang et al., [5] exposed 4-day and 3-month old cultures to PZA for 3 days and found that the 4-day old cultures were four-fold less susceptible to PZA than the stationary phase cultures. Hu et al., [6] also demonstrated that 100-day old static cultures were more susceptible to PZA than 4-day and 30-day old cultures. Our aim was to determine the direct impact of growth rate on the activity of PZA (in a controlled system where the pH is fixed) and ascertain whether growth rate impacts the PZA concentration at which the antibiotic is bacteriostatic or bactericidal against *M. tuberculosis*. We observed populations of either slow growing (constant 69.3 h mean generation time) or fast growing bacilli (constant 23.1 h mean generation time) in their response to the effects of PZA exposure, using controlled and defined growth in chemostats [7, 8].

In vivo, PZA is thought to have a sterilising effect against tubercle bacilli in inflammatory lung lesions, where the pH is acidic (pH 5.5-pH 6.0) [9]. During in vitro studies of PZA activity, the acidity of the growth medium is difficult to control in batch culture and acidified medium is necessary for the susceptibility testing of PZA against *M. tuberculosis.* The optimal pH for the activity of PZA in vitro is pH 5.6 [5]. However, previously it has been shown that *M. tuberculosis* would not grow below pH 5.8 in a hollow fibre model [10], indicating that non-dividing cells are always likely to be the target organism in every PZA susceptibility assay that uses pH 5.6. Previously, we observed that fast growing cells in continuous culture washed out over a period of a few days after the pH was reduced to a controlled acidity level of pH 6.0, the optical density dropped from 2.0_540nm_ down to 0.2_540nm_ within 48 h [11]. The lowest acidity level that *M. tuberculosis* could be maintained at in steady-state growth and at a mean generation time of 23.1 h, was pH 6.2 in chemostat culture. Consequently, a similar pH was used in the current studies to enable *M. tuberculosis* to be grown under controlled environmental conditions in a chemostat at either constant slow or fast growth rates.

The major advantage of continuous culture is that it allows the growth-rate, pH, and oxygen tension to be accurately controlled, and single parameters to be varied independently of each other [7, 8, 12, 13]. Using this approach it was possible to observe the effect of different and controlled growth-rates upon PZA activity in vitro, under defined growth conditions, and at controlled oxygen tension and acidity levels. As shown previously [8], such precise control over environmental conditions is ideal for observing transcriptional responses of *M. tuberculosis* to PZA exposure when grown at different rates. A better understanding of the phenotype of the populations that PZA targets, the in vivo conditions under which it is active, and the mode of action of the drug, will enable us to develop improved assays for testing combinations of antibiotics that include PZA.

## Methods

### Strains, media and antibiotic

*M. tuberculosis* strain H37Rv (NCTC 7416) was used in all experiments. Stock cultures were routinely propagated on Middlebrook 7H10 + OADC agar plates at 37 °C for 3 weeks. Chemostat cultures were grown in CAMR Mycobacterium medium (CMM Mod2) [14]. PZA was purchased from Sigma (P7136) and working stocks of 10 mgml^−1^ were prepared in water and frozen at −20 °C.

### Mycobacterial culture at different growth rates, and exposure to PZA

*M. tuberculosis* (strain H37Rv) was grown in chemostats under controlled conditions as described previously [8] in CMM Mod2 [14], (which contained glycerol as the limiting nutrient), in a culture volume of 500 ml. Two independent continuous cultures were performed at two different growth rates to give a total of four cultures. Mean generation times (MGT) of either 23.1 h (fast growth) or 69.3 h (slow growth) were achieved by manipulating the dilution rates of the culture medium to 0.03 h^−1^ or 0.01 h^−1^, respectively. Steady-state cultures were established under defined and controlled conditions at pH 6.3, at a temperature of 37 °C and at a dissolved oxygen tension of 10 % [8, 12]. pH control was not imposed intially and was commenced when the culture optical density (OD_540nm_) rose above 2.0. Once pH control was initiated, 1 M HCl was added automatically in response to a pH reading higher than the desired set-point. The pH was monitored constantly by an in situ probe (Broadley James). Once the culture density reached the level required, the pH setpoint was gradually lowered by 0.1 pH units per MGT, until pH 6.3 was reached. All cultures were controlled at pH 6.3 by the addition of 1 M HCL, in a steady-state, as defined by turbidity and cell viability, for a minimum of 5 MGT prior to the onset of PZA-addition [11]. PZA was added through the sample port, the medium line was simultaneously drained, and the supply was switched over to medium containing PZA at the appropriate drug concentration. The antibiotic was added to the culture vessels in step-wise increases in the concentration of PZA (25 to 500 μgml^−1^) every 2 MGTs.

### Viability assays

The viabliity of cultures were assessed by preparing decimal dilution series of culture samples in triplicate using sterile phosphate buffered saline as the diluent and plating 100 μL aliquots of each dilution onto Middlebrook 7H10 + OADC plates, in triplicate. The plates were incubated at 37 °C for 4 weeks before colony forming units (cfuml^−1^) were enumerated.

### RNA extraction

For each time-point, 20 ml of culture were harvested for RNA extraction. Extractions and DNase digestion were performed as described previously [8, 12]. Samples were added to 4 volumes of guanidine thiocyanate (GTC) lysis solution (5 M GTC; 5 % lauryl sarcosine; 25 mM Tri-sodium citrate; 0.5 % Tween 80) and incubated at room temperature for 1 h. The sample mixture was centrifuged for 15 min at 1935 × g and the supernatant discarded. The pellets were re-suspended in 1.2 ml of Trizol (Invitrogen 15596018) and mixed thoroughly. The sample was transferred to a 2 ml tube containing 0.5 ml of 0.1 mm silica beads (Fisher Scientific MBR-247-105B) and lysed using a reciprocal shaker (FastPrep FP120) for 45 s at a speed of 6.5. The supernatant was transferred into a tube containing 240 μl of chloroform and shaken vigorously for 20 s. This solution was centrifuged at 2415 × g for 10 min. The aqueous phase was removed and added to 600 μl chloroform, shaken vigorously for 20 s and centrifuged for 10 min at 2415 × g; this process was then repeated. Following the second chloroform precipitation, the aqueous phase was added to 600 μl isopropanol plus 60 μl sodium acetate (Sigma Aldrich S7899) and frozen at −70 °C overnight. Total RNA was isolated from the extractions using the mirVana™ miRNA Isolation kit (Agilent AM1561) and DNase I-treated using the DNA-free™ kit (Ambion® AM1906) as per the manufacturer’s instructions. RNA was quantified using a nanodrop 3000 and the quality assessed on an Agilent 2100 bioanalyzer (Agilent Technologies, CA, USA with an Agilent RNA 6000 Nano Kit (Agilent, 5067-1511).

### RNA labelling

RNA was labelled using the Kreatech ULS™ Fluorescent Labeling Kit for Agilent arrays (Kreatech EA-023) as described previously [8]. The labelled RNA was fragmented by adding 2 μl 10× fragmentation buffer, incubating for 15 min at 70 °C, then adding 2 μl stop solution (Ambion® AM8740). Labelled RNA (20 μl) was added to 27.5 μl Kreatech blocking reagent (Kreatech EA-023), 55 μl of 2× Hybridisation buffer and 7.5 μl of molecular grade water. Arrays were hybridised overnight at 65 °C, then washed in Gene Expression wash buffer 1 (Agilent 5188-5327) for 1 min at room temperature with agitation, then in Gene Expression wash buffer 2 for 1 min at 37 °C with agitation. Slides were scanned immediately using an Agilent Scanner.

### Transcriptomic analyses

Whole genome gene expression analyses were performed. Microarray experiments were performed using a custom Agilent tiling array with 180,000 60-mer oligos evenly tiled across the *M. tuberculosis* H37Rv genome. Features were extracted from the array images using Agilent Feature Extraction Software (v10.7) with local background correction. Probes were first filtered to only include those covering annotated genes. Intensity values were normalised and analysed using GeneSpring software (version 12.6 GX). Firstly, quantile normalisation was applied across the combined slow and fast growth rate datasets, followed by baseline transformation to the median of all samples and averaging of the expression level of all probes across each open reading frame. A 2-way ANOVA (using a p-value cut-off of *P* ≤0.05 and growth rate and MGT as conditions) was used to identify significantly differentially expressed genes. A further filter was applied to select genes with at least a two-fold change in gene expression between time-points of interest. Gene lists derived from all pairwise comparisons can be found in the Additional file 1. Raw data are deposited at ArrayExpress [15] under the accession number E-MTAB-4093.

## Results & discussion

### The effect of growth rate on the sensitivity of *M. tuberculosis* to PZA

**Fig. 1:**
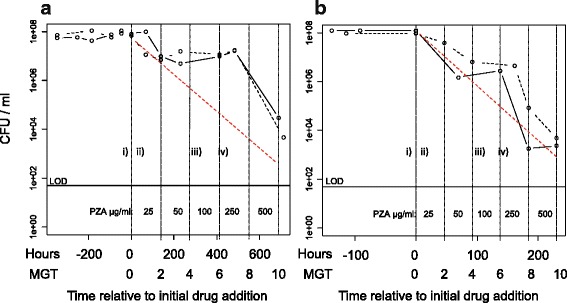
The viability of *M. tuberculosis* H37Rv at either a fast growth rate or a slow growth rate in response to PZA exposure. Viability of *Mycobacterium tuberculosis* H37Rv growing at either a slow growth rate (69.3 h mean generation time (MGT); Panel **a** or a fast growth rate (23.1 h MGT; Panel **b** The cultures were exposed to sequential increases in the concentration of PZA in a step-wise manner every 2 MGTs and bacterial survival was measured. The levels of PZA used were 25, 50, 100, 250 and 500 μgml^−1^; these concentrations are indicated on each graph between the vertical dotted lines to show each exposure period. Each growth rate was cultured in duplicate (solid and dashed black lines). The washout rate of the culture system is represented by the gradient of the dashed red lines. A reduction in bacterial number (cfuml^−1^) that followed this rate was indicative of a bacteriostatic drug effect. A more rapid rate of loss of viable cells indicated a bactericidal effect, and a less rapid rate indicates a sub-inhibitory effect. The limit of detection (LOD; defined as the level calculated from the presence of a single colony in one technical replicate) is indicated by the line labelled LOD. The time-points for which transcriptional analyses were performed are indicated as follows: i) pre-PZA phase, ii) bacteriostatic phase (4 h after the addition of 25 μgml^−1^) iii) early recovery phase (4 h after the addition of 100 μgml^−1^ PZA) and iv) late recovery phase (2 MGT after the addition of 100 μgml^−1^ PZA)

*M. tuberculosis* cultured at a fast growth rate showed a similar response to increasing concentrations of PZA as slow growing bacilli. Duplicate cultures at either a fast growth rate (MGT of 23.1 h) or slow growth rate (MGT of 69.3 h) achieved a steady state in which the total viable cell numbers remained constant at 1 × 10^8^ cfuml^−1^ and 7 × 10^7^ cfuml^−1^, respectively, for a minimum of 5 MGT, at a controlled pH of 6.3 (Fig. 1).

The cultures were exposed to sequential increases in the concentration of PZA in a step-wise manner every 2 MGTs and bacterial survival was measured. The levels of PZA used were 25, 50, 100, 250 and 500 μgml^−1^ (Fig. 1). The washout rate of the chemostat (the theoretical rate at which non-dividing cells are removed from the culture by dilution) is indicated in Fig. 1 by the red dotted line. A reduction in the bacterial numbers that followed this rate was indicative of a bacteriostatic drug effect. A more rapid rate in the loss of bacteria indicated a bactericidal effect, and a less rapid rate indicates a sub-inhibitory effect.

Actively dividing mycobacteria were susceptible to PZA, and slow and fast growing bacteria were affected similarly (Fig. 1a & b, respectively). Both fast and slow growing bacilli showed an approximately log-fold reduction in bacterial numbers following exposure to just 25 μgml^−1^ PZA, which equated to an approximately 90 % decrease in cfuml^−1^ after 2 MGT (Fig. 1), after which time the growth rate was re-established. The rate of intial loss of bacterial numbers from the system was of a similar magnitude to the washout rate and was indicative of a bacteriostatic drug effect and a minimum inhibitory concentration (MIC) of ≤25 μgmL^−1^. This MIC agrees with previous findings by Gumbo et al., in 2009 [10] using the hollow fibre. Curiously, they deduced a similar MIC at a lower level of acidity of pH5.8. They also observed a lack of killing for four days post PZA-exposure.

Following the increase in PZA concentration to 50 μgml^−1^, a plateauing in cell titre occurred in the slow growth rate cultures, at ~ 1 × 10^7^cfuml^−1^ (Fig. 1a, approximately 10 % of pre-antibiotic viable cell levels). The slow growers had adapted to the presence of PZA and remained in steady-state for 4 MGT through a further increase in PZA to 100 μgml^−1^. The fast growth-rate cultures continued to lose viability during exposure to 50 μgml^−1^ but then followed a similar but delayed response by plateauing in their cell number between 100 and 250 μgml^−1^ PZA (Fig. 1b). When the PZA concentration reached 250 μgml^−1^ the bacterial numbers fell in the fast growing cultures (Fig. 1b) to approximately 1 % of original pre-PZA levels after 2 MGTs, which continued following the increase to 500 μgml^−1^. It is not possible for us to say whether a PZA concentration of 250 μgml^−1^ was also bactericidal for the slow growth cultures (Panel a, Fig. 1) as after one MGT at that concentration the bacilli in these cultures were still dividing. Death of the slow growers was observed between 7MGT and 10 MGT. However, this may have been from exposure to a PZA concentration of 500 μgmL^−1^ and not 250 μgmL^−1^. A time-point at 8 MGT was included for the fast growers but not the slow growers. This time-point provides critical information for us to be able to conclude that bacilli at each growth rate were sterilised by the same concentration of PZA. It appears that the fast growers could have been killed at a lower PZA concentration than the slow growers but further time-courses would need to be performed to be certain of this. By the end of the PZA-exposure time-course (10 MGT), less than 0.1 % of the original viable cell population was remaining in all cultures. The results show that high concentrations of PZA will sterilise the population irrespective of growth rate. The bactericidal response observed in our cultures was at a concentration that was much higher than observed previously in studies when the environmental pH was lower. The experiments described here were designed to specifically determine the effect of growth rate on PZA efficacy and therefore a higher pH had to be used to enable cells to continue to divide.

The mode of action of PZA was described previously by Zhang et al., in 2003 [16]. PZA, a pro-drug, is converted to pyrazinoic acid (POA) by an amidase (encoded by gene; Rv2043c, *pncA*) found in the bacterial cytoplasm [17]. POA is excreted from the bacteria and then it diffuses passively back in through the cell wall as the protonated molecule (HPOA) when the pH of the bacterial environment is acidic. Multiple mechanisms of action have been identified for POA; it has been shown to decrease the proton motive force and ATP synthesis rates in mycobacterial membranes as well as lowering cellular ATP levels in *Mycobacterium bovis* BCG [18]. Further support is given to this by the observations that some weak acids and energy inhibitors work synergistically with PZA [19] and mutations in energy production and ion homeostasis pathways enhance PZA activity [20]. The reported bactericidal concentration of PZA in vitro under more acidic condtions at a pH of 5.6 is 50 μg/ml [5]. Bactericidal effects were observed between 250 and 500 μgml^−1^ PZA in this study for both growth rates; this reduction in effectiveness (5-fold) is in proportion with the expected fold-decrease in internal POA concentration commenserate with a less acidic external condition in our chemostat cultures (pH 6.3) as calculated using the Henderson-Hasselbalch equation [5, 21]. Previously, it had been shown that the slowing of bacterial growth reduced the activity of an efflux pump, which is then inefficient at extruding HPOA from the cell, thereby leading to an accumulation of HPOA and subsequently increased bactericidal effects. In our study, we did not observe any effect of bacterial growth rate on the overall sterilising activity of PZA. This suggests that it is not growth rate *per se* that determines PZA activity; increased drug activity observed during stationary phase must be due to other aspects of bacterial metabolism.

PZA has no detectable early bactericidal activity (EBA) in the first 2 days of treatment [22]; monotherapy ranges from 0.04 to 0.1 log_10_cfuml^−1^day^−1^ [9]. However, despite this lack of efficacy, PZA exerts an effect by shortening treatment only during the first 2 months of treatment and not beyond this. Extending the duration of treatment with PZA has no additional benefit in either humans or murine models [3, 23, 24]. Can we be sure that PZA is not exerting a profound bacteriostatic effect against actively dividing organisms during the first phase of treatment? It has always been assumed that INH clears the fast growing population during EBA leaving slow growing drug-tolerant (as opposed to genetically resistant) bacteria that need to be cleared by other drugs such as PZA. However, in a recent study we showed that both fast and slow growing cells persist through INH exposure via different growth rate-specific genotypic and phenotypic mechanisms [8]. Therefore, we cannot generalise that fast growing bacteria are sterilised during EBA and slow growing cells are sterilised later during treatment. The contribution of PZA to the clearance of fast growing bacteria during the first phase of treatment has not been fully explored but it may be having hidden and profound effects such as controlling bacterial numbers or preventing antibiotic resistance from arising. Could the activity of PZA be improved during the first phase of treatment by using it in combination with other antibiotics that boost its activity? The maximum peak level of PZA attainable in patients’ blood is 30–60 μgml^−1^ which is the optimal killing concentration in vitro at pH 5.6 [25]. More defined and reproducible in vitro systems that can test combinations of drugs, including PZA, against *M. tuberculosis* and achieve lower concentrations of PZA which deliver more potent bactericidal activity would help to shorten treatment times. Previously, Grosset et al., [26] demonstrated in mice that INH could be having antagonistic effects on the efficacy of PZA, and that there might be benefits of removing INH after the first 2 days of treatment. As PZA is a lead drug in terms of good sterilising activity, it is important to understand how other drugs have an impact on PZA efficacy and how we can rationalise the use of current drugs to optimise its activity. The postponement of PZA use after the initial bactericidal phase has been suggested [26] but this may not be beneficial given the information presented here demonstrating that PZA has a comparable level of activity against fast and slow growing cells of *M. tuberculosis*. It may be more appropriate to optimise combinations that improve the efficacy of PZA from the start of treatment. Is there a case for replacing INH with alternative antibiotics that increase the activity of PZA and do not give rise to resistance in the way that INH does? This has been demonstrated recently by a clinical trial of PZA in combination with moxifloxacin and Pretomanid (PA-824) [27], which showed superior bactericidal activity in drug-susceptible TB during 8 weeks of treatment. Results were consistent between drug-susceptible and multi-drug resistant (MDR) TB. This new regimen is ready to enter phase 3 trials in patients with drug-susceptible TB and MDR-TB, with the goal of shortening and simplifying treatment.

### Transcriptional analysis

**Fig. 2:**
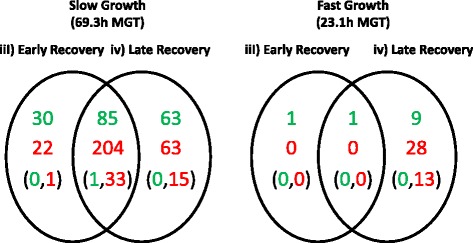
Significantly differentially expressed genes during PZA-exposure. Numbers of genes up-regulated (in *green*) or down-regulated (in *red*), with a fold-change greater than two-fold and ANOVA *p*-value less than 0.05, in the early recovery phases (4 h after the addition of 100 μgml^−1^ PZA) and late recovery phases (2 mean generation times (MGT) after the addition of 100 μgml^−1^ PZA) compared to the pre-PZA phases under fast growth rates (23.1 h MGT) and slow growth rates (69.3 h MGT). The numbers shown in brackets refer to the subset of genes in each case that encode either tRNAs or ribosomal protein components

Three distinct phases of response to PZA can be distinguished in cells growing at slow or fast growth rates (Fig. 1, panels a & b, respectively) There was an initial loss of bacteria as PZA was added to the system at inhibitory levels (25 μgml^−1^; “bacteriostatic phase”), followed by a phase in which bacilli had adapted to the presence of PZA and were actively dividing in steady state (“recovery phase”). Finally a rapid loss of culture viability was observed once the levels of PZA became bactericidal (“bactericidal phase”). The most notable difference in the response to PZA between cells growing at the two growths rates was that the slow growing bacteria plateaued earlier in the recovery phase than the fast growers (Fig. 1). Global gene expression was analysed to see if the mechanisms of adaptation to pyrazinamde in the ‘recovery phase’ were different between bacilli cultured at different growth rates. RNA was extracted at time-points prior to PZA-addition (“pre-PZA phase” Fig. 1.i)), during the bacteriostatic phase (4 h after the addition of 25 μgml^−1^, “bacteriostatic phase” Fig. 1.ii)), and during the recovery period (4 h after the addition of 100 μgml^−1^ PZA, “early recovery phase” Fig. 1.iii), and 2 MGT after the addition of 100 μgml^−1^ PZA, “late recovery phase” Figure.iv)). The RNA was analysed by whole genome DNA microarray and the data are deposited in ArrayExpress, accession no. E-MTAB-4093 [15]. The two time-points within the recovery phase were selected to cover the overlap in the recovery periods for the two growth rates. The 4 h timepoint was early on in the recovery of the fast growers and mid-recovery for slow (“early recovery” Fig. 1.iii)), whereas the time-point at 2MGT after this was at the end of the recovery period for both growth rates (“late recovery” Fig. 1.iv)). No genes were differentially expressed between the pre-PZA phase (Fig. 1.i)) and the bacteriostatic phase (Fig. 1.ii)) under slow growth using selection criteria of an Anova p-value of less than 0.05 and a minimum fold change of two. In contrast, four genes were up-regulated under fast growth between these phases: Rv2645 (uncharacterised), Rv2738c (uncharacterised), Rv3221 (biotynylated protein TB7.3), and Rv3018c (uncharacterised PPE family protein), (Additional file 1: Table S1). Following this, comparisons were made between the pre-PZA phase and the recovery phases. There were 341 differentially expressed genes in the early recovery phase (Fig. 1.iii)) compared to pre-PZA phase (Fig. 1.i)) in slow growing cells, which consisted of 115 up-regulated genes and 226 down-regulated genes (Fig. 2. & Additional file 1: Table S2). In the late-recovery phase (Fig. 1.iv)) there were 415 genes differentially expressed under slow growth, which consisted of 148 up-regulated genes and 267 down-regulated genes (Fig. 2. & Additional file 1: Table S2). There were 85 shared up-regulated genes and 204 shared down-regulated genes across the two timepoints in the recovery phase (Fig. 1 iii) and iv)) for slow growth (Fig. 2. & Additional file 1: Table S2.). In the fast-growing cultures there were only two differentially expressed genes in the early recovery phase (Fig. 1.iii)), both of which were up-regulated. In the late recovery phase (Fig. 1iv)), 38 genes were differentially expressed, which consisted of 10 up-regulated genes and 28 down-regulated genes (Fig. 2. & Additional file 1: Table S3).

### Differentially expressed genes were enriched for functions encoding Ribosomal proteins and tRNAs

To determine whether differentialy expressed genes were significantly enriched for particular functions, functional annotation clustering reports were generated using the DAVID 6.7 bioinformatic resource [28] with Uniprot IDs as the input. A Bonferroni-corrected EASE-value cut-off of 0.01 (EASE-value being an alternative name for the one-tail Fisher Exact Probability Value used to measure gene-enrichment in annotation terms, compared to proportions of these genes encoded within the genome) was employed along with a minimum gene number per category of five genes (i.e. a category cannot be considered to be enriched unless at least five genes from a function are present). The functional annotation clustering report groups similar categories of annotations together into clusters. None of the up-regulated genes were significantly enriched for any functional categories. Under slow growth, the 226 down-regulated genes in the early recovery phase and the 267 down-regulated genes in the late recovery phase (Fig. 2.) were significantly enriched in 15 categories as defined by the GO ontology database [29], the Protein Information Resource (PIR) [30], and the KEGG pathway database [31] (Table 1.). All 15 categories were clustered into a single group by DAVID, and no other categories outside the group were enriched. All categories relate to ribosomal functions. From the fast growth rate cultures, there were no significantly enriched categories in the early recovery phase, but the 31 down-regulated genes in the late recovery phase (Fig. 2.) showed significant enrichment for the same 15 ribosomal function related categories seen under slow growth conditions.

**Table 1:** DAVID Functional annotation clustering of genes that were down-regulated in *M. tuberculosis* exposed to PZA under different growth rates in early and late recovery

		Slow growth (69.3 h MGT)				Fast growth (23.1 h MGT)			
		Down-regulated in early recovery		Down-regulated in late recovery		Down-regulated in early recovery		Down-regulated in late recovery	
Annotation scheme	Annotation	Count	Bonferroni corrected EASE-value	Count	Bonferroni corrected EASE-value	Count	Bonferroni corrected EASE-value	Count	Bonferroni corrected EASE-value
KEGG_PATHWAY	ribosome	25	4.00E-24	28	1.70E-26	n/a	n/a	7	4.20E-08
GOTERM_MF_FAT	structural constituent of ribosome	25	1.60E-21	28	4.10E-25	n/a	n/a	7	1.10E-08
SP_PIR_KEYWORDS	ribonucleoprotein	25	3.60E-21	28	3.40E-24	n/a	n/a	7	1.90E-06
GOTERM_MF_FAT	rRNA-binding	21	1.00E-20	23	2.30E-23	n/a	n/a	4	1.10E-03
SP_PIR_KEYWORDS	rRNA-binding	21	7.80E-21	23	4.30E-23	n/a	n/a	4	8.10E-03
SP_PIR_KEYWORDS	ribosomal protein	25	3.50E-20	28	5.00E-23	n/a	n/a	7	3.10E-06
GOTERM_MF_FAT	structural molecule activity	25	5.50E-20	28	2.60E-23	n/a	n/a	7	2.50E-08
GOTERM_CC_FAT	ribosome	25	2.50E-19	28	1.30E-21	n/a	n/a	7	9.80E-05
GOTERM_CC_FAT	ribonucleoprotein complex	25	8.80E-19	28	5.60E-21	n/a	n/a	7	1.30E-04
GOTERM_BP_FAT	translation	29	1.10E-17	32	1.00E-19	n/a	n/a	7	9.20E-06
SP_PIR_KEYWORDS	RNA-binding	22	7.50E-16	25	1.50E-18	n/a	n/a	4	4.50E-02
GOTERM_MF_FAT	RNA binding	26	1.80E-14	27	1.80E-14	n/a	n/a	5	1.50E-03
GOTERM_CC_FAT	ribosomal subunit	10	8.60E-09	10	3.60E-08	n/a	n/a	4	3.20E-03

A corrected EASE-value of 0.01 and minimum gene number per category of five was used to perform functional annotation clustering on all down-regulated genes in the early recovery phase (4 h after the addition of 100 μgml^−1^ PZA) or the late recovery phase (2 MGT after the addition of 100 μgml^−1^ PZA) compared to the pre-PZA phase

At a gene-specific level, during the early recovery phase, eight tRNA-encoding genes and 26-ribosomal-protein encoding genes were down-regulated in the slow growing cultures (Table 1, Fig. 2. brackets, Table 2.), with a single up-regulated gene encoding a ribosomal protein (Rv2442, 50s ribosomal protein L21). In contrast, no genes of this function were differentially expressed in the fast growing cultures. By late recovery under slow growth, there were 17 tRNA-encoding genes and 31 ribosomal-protein encoding genes showing significant down-regulation compared to the pre-PZA phase. There were also 6 tRNA-encoding genes and seven ribosomal protein encodoing genes down-regulated in the fast growing bacteria in the late recovery phase (Table 3.). The delayed down-regulation seen during fast growth was co-incident with the delayed plateau in culture viability.

**Table 2:** Ribosomal and tRNA associated genes down-regulated in *M. tuberculosis* exposed to PZA under slow growth

Gene expression fold change from pre-PZA to early recovery phase (100 μgml^−1^ for 4 h)	Gene expression fold change from pre-PZA to iv) late recovery phase (100 μgml^−1^ for 2 MGT)	Gene name	UniProt ID	Annotation
−4.81	−11.22	tRNA-Val		tRNA-Val
−3.23	−10.01	tRNA-Glu		tRNA-Glu
−3.99	−9.34	tRNA-Lys		tRNA-Lys
−5.13	−8.95	Rv0700	P0A5X0	30S ribosomal protein S10
−2.63	−8.62	tRNA-Gly		tRNA-Gly
−3.52	−8.60	tRNA-Thr		tRNA-Thr
−4.86	−8.15	tRNA-Arg		tRNA-Arg
−5.22	−7.36	Rv0704	P95052	50S ribosomal protein L2
−4.49	−7.33	tRNA-Met		tRNA-Met
−4.68	−6.35	Rv0703	P95051	50S ribosomal protein L23
−4.29	−6.16	Rv1298	P66187	50S ribosomal protein L31
−1.65	−6.13	tRNA-Leu		tRNA-Leu
−2.33	−5.97	tRNA-Ser		tRNA-Ser
−4.08	−5.88	Rv0710	P95058	30S ribosomal protein S17
−1.65	−5.43	tRNA-Pro		tRNA-Pro
−3.70	−5.20	Rv0705	P0A5X4	30S ribosomal protein S19
−1.44	−4.83	tRNA-Tyr		tRNA-Tyr
−3.56	−4.82	Rv0709	P95057	50S ribosomal protein L29
−4.32	−4.82	Rv0702	P60729	50S ribosomal protein L4
−3.99	−4.72	Rv0722	P66181	50S ribosomal protein L30
−3.09	−4.66	Rv0701	P60442	50S ribosomal protein L3
−3.76	−4.17	Rv0716	P62403	50S ribosomal protein L5
−3.32	−3.98	Rv0717	P0A5X2	30S ribosomal protein S14 type Z
−3.48	−3.79	Rv0682	P41196	30S ribosomal protein S12
−3.01	−3.69	Rv0715	P60627	50S ribosomal protein L24
1.03	−3.68	tRNA-Cys		tRNA-Cys
−3.45	−3.61	Rv1643	P66105	50S ribosomal protein L20
−2.99	−3.56	Rv0723	P95071	50S ribosomal protein L15
−1.44	−3.38	tRNA-Trp		tRNA-Trp
−2.97	−3.32	Rv2412	P66505	30S ribosomal protein S20
−3.07	−3.22	Rv0683	P41194	30S ribosomal protein S7
−4.07	−3.05	Rv0718	P66625	30S ribosomal protein S8
−3.13	−3.04	Rv1642	P66271	50S ribosomal protein L35
−2.59	−3.02	Rv0714	P66069	50S ribosomal protein L14
−2.79	−2.92	Rv0707	P0A5X6	30S ribosomal protein S3
−1.06	−2.91	5S_rRNA		ribosomal RNA
−2.44	−2.91	Rv0721	P66574	30S ribosomal protein S5
1.03	−2.84	tRNA-Asp		tRNA-Asp
−1.14	−2.67	tRNA-His		tRNA-His
−1.10	−2.65	tRNA-Phe		tRNA-Phe
−1.45	−2.64	tRNA-Ile		tRNA-Ile
1.00	−2.45	23S rRNA		ribosomal RNA
−1.41	−2.39	Rv0640	P66056	50S ribosomal protein L11
1.00	−2.27	16S rRNA		Ribosomal RNA
−2.01	−2.27	Rv0720	P66076	50S ribosomal protein L18
−1.84	−2.17	Rv0719	P66311	50S ribosomal protein L6
−2.15	−2.07	Rv0055	P69230	30S ribosomal protein S18 1
−1.67	−2.03	Rv1630	O06147	30S ribosomal protein S1
−2.35	−1.92	Rv1644	P94978	possible 23S rRNA methyltransferase tsnR

Genes encoding tRNAs or ribosome component proteins that were down-regulated by at least two fold (with an ANOVA *p* –value of less than 0.05) in *M. tuberculosis* under slow growth in the early recovery phase (4 h after the addition of 100 μgml^−1^ PZA) and/or the late recovery phase (2 MGT after the addition of 100 μgml^−1^ PZA) compared to the pre-PZA phase

**Table 3:** Ribosomal and tRNA associated genes down-regulated in *M. tuberculosis* exposed to PZA under fast growth

Fold change from pre-PZA to early recovery phase (100 μgml^−1^ for 4 h)	Fold change from pre-PZA to late recovery phase (100 μgml^−1^ for 2 MGT)	Gene name	UniProt ID	Annotation
1.12	−2.03	tRNA-Glu		tRNA-Glu
−1.45	−2.07	Rv0704	P95052	50S ribosomal protein L2
−1.43	−2.10	Rv0723	P95071	50S ribosomal protein L15
−1.02	−2.11	tRNA-Thr		tRNA-Thr
−1.07	−2.20	tRNA-Lys		tRNA-Lys
−1.12	−2.26	tRNA-Arg		tRNA-Arg
−1.55	−2.31	Rv0703	P95051	50S ribosomal protein L23
−1.60	−2.33	Rv0700	P0A5X0	30S ribosomal protein S10
−1.51	−2.40	Rv0722	P66181	50S ribosomal protein L30
−1.14	−2.42	tRNA-Val		tRNA-Val
−1.79	−2.45	Rv0709	P95057	50S ribosomal protein L29
−1.90	−2.72	Rv0710	P95058	30S ribosomal protein S17
−1.10	−2.04	tRNA-Met		tRNA-Met

Genes encoding tRNAs or ribosome component proteins that were down-regulated by at least two fold (with an ANOVA *p* –value of less than 0.05) in *M. tuberculosis* under fast growth in the early recovery phase (4 h after the addition of 100 μgml^−1^ PZA) and/or the late recovery phase (2 MGT after the addition of 100 μgml^−1^ PZA) compared to the pre-PZA phase

It is challenging to ascertain which of the PZA targets make the biggest contribution to cell death. With so many modes of action does PZA act predominantly upon the the same target and in all bacterial phenotypes under different growth conditions? Given the multiple modes of action, we were interested in which phenotypic mechanisms were employed by fast and slow growing bacilli to enable them to overcome the effects of the antibiotic and to observe any obvious growth-rate specific mechanisms. It was postulated previously that POA disrupts bacterial membrane energetics and inteferres with energy production [16]. Stationary phase bacilli might have a lower membrane potential than actively dividing log-phase bacteria, which could explain the increased activity of PZA against stationary phase bacteria observed previously. No gene expression changes were observed for genes encoding functions related to membrane potential. Bacilli in fast and slow growth cultures may not have exhibited much difference in membrane potential explaining the similar response to PZA-exposure. Understanding the specific differences between bacterial stationary phase and slow growth might enable us to dissect out the range of activities that PZA has against bacilli in different phenotypic states. Fast growers and slow growers were adapting to PZA-exposure via very similar mechanisms. The only functions that were differentially expressed under each growth rate, that were also enriched for function using DAVID analyses, were genes encoding tRNA, 50S and 30S ribosomal proteins, which were down-regulated during the recovery phase. This may go some way to explaining why the populations at both growth rates were adapting and establishing new steady-states, but at a lower cell density. The maximum cell number sustainable on the available nutrient was simply less because of a reduction in level of protein that was being translated leading to a subsequent decrease in available target. Recently, Shi et al., [32] found that POA binds the ribosomal protein S1 (RspA), which is a vital protein for trans-translation, and inhibits the trans-translation activity required for efficient protein synthesis. Mutations in *rpsA* (gene Rv1630) result in PZA-resistance, and are alternative mechanisms of resistance to mutations in *pncA* [33–35]*.* Trans-translation is essential for rescuing stalled ribosomes during translation in non-replicating organisms and inhibition of this process could be one mechanisms by which PZA targets persisting organisms. Bacteria require *trans*-translation to respond to stress, pathogenesis, and differentiation, which indicates that fast growing organisms also require *trans*-translation to overcome stress [36]. Based on this mode of action for PZA, it follows that fast-growing organisms would be targeted by PZA. Increased PZA efficacy against fast growing cells could be enabled by potentiators that create stress/reduce metabolism, such as energy inhibitors, in fast growers [37, 38].

The recovery period co-incident with the down-regulation of ribosomal protein encoding genes seen in this study might reflect a mechanism to reduce the production of toxic proteins caused by the inhibition of trans-translation [39]. Alternatively adaptation may have occurred through an increase in the mutant frequency and diversity of mutations as previously shown in the adaptation of fast and slow growing *M. tuberculosis* to INH exposure and we would expect (as shown for INH-exposure) that there would be a difference in the mutant profile between the two different growth rates. Much is understood about how *M. tuberculosis* adapts to PZA-exposure by the development of resistance mutations in *pncA* and more recently mutations have been shown in *panD* [20], which encodes aspartate-alpha-decarboxylase involved in the synthesis of β-alanine that is a precursor for pantothenate and co-enzyme-A biosynthesis [40] and may be yet another target for PZA.

## Conclusions

PZA has an equivalent level of activity against fast growing and slow growing *M. tuberculosis* under the conditions tested here. Both populations appeared to adapt to PZA*-*exposure via reduced expression of tRNA, 50S, and 30S ribosomal proteins. Understanding the subtle differences between bacterial growth rate and growth phase will enable us to dissect out the range of activities that PZA has against bacilli in different phenotypic states and aid the development of drug combinations containing PZA. The contribution of PZA to the clearance of actively dividing bacilli during the first phase of treatment has not been fully explored. However, the findings from this study show that PZA has utility against fast growing bacteria and it demonstrates that the assessment of drug combinations containing PZA, used early in treatment, warrants further investigation.

### Ethics approval and consent to participate

Not applicable.

### Consent for publication

Not applicable.

### Availability of data and materials

The datasets supporting the conclusions of this article are available in the ArrayExpress repository at https://www.ebi.ac.uk/arrayexpress/experiments/E-MTAB-4093/

Data are also included in the Additional file 1.

## Additional file

Additional file 1: Supplementary tables, S1, S2 and S3. Differentially regulated transcripts in the fast cultures during bacteriostatic and recovery phase compared to pre-PZA phase, and in the slow cultures during recovery phase compared to pre-PZA phase. (XLSX 55 kb)

## Abbreviations

CMM Mod2: CAMR mycobacterium medium
EBA: early bactericidal activity
GTC: guanidine thiocyanate
INH: isoniazid
*M. tuberculosis*: *Mycobacterium tuberculosis*
MDR: multi-drug resistance
MGT: mean generation time
MIC: minimum inhibitory concentration
OD: optical density
POA: pyrazinoic acid
PZA: pyrazinamide
TB: tuberculosis
